# Supplementary material for: Rituximab-Containing Treatment Regimens May Imply a Long-Term Risk for Difficult-To-Treat Chronic Hepatitis E
Source: Int J Environ Res Public Health. 2020 Jan 3;17(1):341. doi: 10.3390/ijerph17010341 (PMC6982013; doi:10.3390/ijerph17010341)
Supplement: Supplementary file 1 [file ijerph-17-00341-s001.pdf]

Table S1. Reported cases of hepatitis E after administration of RTX:.

	underlying disease	time from last RTX administration to HEV diagnosis	immunosuppressive medication	HEV treatment	treatment outcome	n
**von Felden et al. 2019 [9]**	n/d	n/d	RTX/bendamustine or R-CHOP	n/d	n/d	11
**Bauer et al. 2015 [12]**	rheumatoid arthritis<br>rheumatoid arthritis<br>rheumatoid arthritis<br>rheumatoid arthritis	n/d	RTX/leflunomide/MTX<br>RTX/MTX<br>RTX/leflunomide/steroids<br>RTX/MTX	discontinuation of leflunomide/MTX<br>3 months of RBV<br>discontinuation of leflunomide<br>MTX replaced by leflunomide	n/d<br>HEV clearance<br>n/d<br>n/d	4
**Fraticelli et al. 2016 [13]**	sjögren's syndrome	9 months	RTX/MMF	discontinuation of RTX/MMF<br>3 months of RBV 2x400 mg/d	relapse	1
**Gauss et al. 2012 [14]**	B-cell lymphocytic leukemia	6.5 months	RTX/pentostatin/cyclophosphamide	discontinuation of RTX	HEV clearance	1
**Alnuaimie et al. 2018 [15]**	indolent lymphoma<br>indolent lymphoma	12 months<br>7 months	RTX/bendamustine<br>RTX/bendamustine	2 cycles of RBV<br>4 months of RBV (10mg/kg/d)	death<br>relapse	2
**Verhoeven et al. 2016 [16]**	rheumatoid arthritis	7 months	RTX	2 months of RBV 2x800mg/d	HEV clearance	1
**Ollier et al. 2009 [17]**	non-Hodgkin lymphoma	n/d	RTX/chlorambucil/fludarabine	3 months interferon	HEV persistance	1

Abbreviations: R-CHOP – rituximab, cyclophosphamide, doxorubicin, vincristine, prednisolone; MTX - methotrexate; n/d – not determined; MMF - mycophenolate mofetil
